# Microsatellite Investigations of Multiple *Echinococcus granulosus* Sensu Stricto Cysts in Single Hosts Reveal Different Patterns of Infection Events between Livestock and Humans

**DOI:** 10.3390/pathogens9060444

**Published:** 2020-06-05

**Authors:** Selim M’rad, Myriam Oudni-M’rad, Vanessa Bastid, Laure Bournez, Sana Mosbahi, Abdelallatif Nouri, Hamouda Babba, Frédéric Grenouillet, Franck Boué, Gérald Umhang

**Affiliations:** 1 Laboratory of Medical and Molecular Parasitology-Mycology (LP3M), LR12ES08, Faculty of Pharmacy, University of Monastir, Monastir 5000, Tunisia; selim.mrad@gnet.tn (S.M.); myriam.mrad@gnet.tn (M.O.-M.); hamouda.babba@gnet.tn (H.B.); 2 Anses LRFSN, Wildlife Surveillance and Eco-Epidemiology Unit, National Reference Laboratory for *Echinococcus* spp., 54220 Malzéville, France; vanessa.bastid@anses.fr (V.B.); laure.bournez@anses.fr (L.B.); franck.boue@anses.fr (F.B.); 3 Paediatric Surgery Department, F. Bourguiba Hospital, Monastir, Medical School, Monastir 5000, Tunisia; sana_mosbahi@yahoo.fr (S.M.); drnouri.abdellatif@gmail.com (A.N.); 4 Laboratory of Parasitology-Mycology, EPS F. Bourguiba, Monastir 5000, Tunisia; 5 Chrono-Environnement UMR 6249 Research Team, CNRS-University of Bourgogne-Franche-Comté, 25030 Besançon, France; fgrenouillet@chu-besancon.fr; 6 Medical Biology Department, and French National Reference Centre for Echinococcosis, University Hospital of Besançon, 25030 Besançon, France

**Keywords:** cystic echinococcosis, *Echinococcus granulosus* sensu stricto, microsatellite, intra-individual genetic diversity, infection event, recurrence

## Abstract

Cystic echinococcosis (CE) caused by the cestode *Echinococcus granulosus* sensu lato (s.l.) is a worldwide zoonosis and *E. granulosus* sensu stricto (s.s.) is the most common species associated with animal and human diseases. The objective of this study was to obtain a better understanding of CE infection in livestock and humans from very low and high endemic areas—France and Tunisia—by studying the genetic diversity of *E. granulosus* s.s. at the intra-individual host level. This genetic diversity was studied using EgSca6 and EgSca11 microsatellite profiles in 93 sheep from France and Tunisia, and in 12 cattle and 31 children from Tunisia only, all presenting multiple CE cysts (2 to 10 cysts). Overall, 96% of sheep, 92% of cattle, and 48% of children had at least two cysts with different microsatellite profiles. Inversely, 35% of sheep, 17% of cattle, and 65% of children had at least two cysts with the same microsatellite profile. The genotyping results for the CE samples highlight high and similar genetic diversity in France and Tunisia, suggesting that the probability of being successively infected by CE of the same microsatellite profile was rare in both countries. Therefore, our results suggest that in rare cases, several eggs of the same microsatellite profile, from two to seven in our data, can be ingested simultaneously in a single infection event and develop into several cysts in livestock and children. They also indicate that multiple infection events are frequent in livestock, even in a low endemic country such as France, and are less frequent but not negligible in children in a high endemic country such as Tunisia. Moreover, this is the first time that genetic evidence of secondary CE has been found. Further studies are needed to better assess the pattern of infection events in livestock and humans, especially by studying the genetic diversity of adult worms in definitive hosts.

## 1. Introduction

Cystic echinococcosis (CE), previously known as hydatidosis, caused by the cestode *Echinococcus granulosus* sensu lato (s.l.) is a worldwide zoonosis. CE is frequently encountered in countries where pastoral livestock breeding is well developed [1]. With a mean annual surgical incidence (SI) of 12.6/100,000 inhabitants, Tunisia is considered the most endemic Mediterranean area [2]. In European Mediterranean countries such as France or Italy, the SI is much lower and averages 0.42/100,000 and 1.6/100,000 inhabitants, respectively [3,4,5]. The lifecycle involves a final host, generally a canid, harboring the adult parasite stage and an herbivore intermediate host, harboring the parasite larval stage. Humans are considered accidental hosts. Human and herbivore infections are acquired through the oral ingestion of *E. granulosus* s.l eggs via vegetables and water, or for humans by direct contact with dogs [6]. The eggs hatch in the bowels and release oncospheres that penetrate the intestinal wall, before migrating through the circulatory system. Each viable oncosphere has the potential to develop into one CE cyst (larval stage), which is most commonly located in the liver and/or the lungs even though almost any organ may be affected [7].

Five species are currently recognized as responsible for CE: *E. granulosus* sensu stricto (s.s.), *E. equinus*, *E. ortleppi*, *E. canadensis,* and *E. felidis* [8,9,10,11]. *Echinococcus granulosus* s.s. (particularly the G1 genotype) is the most common CE-causing species associated with ovine, bovine and human CE [12,13,14]. The genetic diversity of *E. granulosus* s.s. at the intra-specific level is usually assessed by comparison of haplotypes from fragments, full lengths of mitochondrial genes (e.g., *cox1* and *nad1*), or near-complete mitogenome sequences. Results have revealed high diversity of *E. granulosus* s.s. haplotypes in several parts of the world [11,15,16,17,18,19,20,21,22]. Overall, all the studies have highlighted distribution of *E. granulosus* s.s. shaped by the spread of livestock domestication and intensive animal trade.

Although studies have also proven that genetic diversity may vary depending on the host [23,24] and/or the larval localization [25,26], genetic diversity in a single host has rarely been investigated by molecular tools. Though generally overlooked, the study of genetic diversity at the intra-individual host level appears to be a useful approach to better understand CE infection events (i.e., ingestions of eggs, at the same or different moment, leading to the development of one or more CE cysts). The possibility of genetically identifying each cyst in a single infected host would make it possible to estimate the number of successful infection events that this host has encountered. This is an important consideration in terms of public health. In fact, among human CE cases, the proportion of children in Tunisia having several cysts is estimated to be around 20% [27,28]. However, it is unknown whether these multiple cysts result from a single or successive infection events. It might then also be possible to compare the number of infection events in humans and livestock, bearing in mind that a priori, the exposure of herbivores to environmental contamination by eggs of *E. granulosus* s.s. is expected to be much higher. As the frequency of infection events is strongly linked to the level of environmental contamination by eggs, different patterns of infection may be suspected between very low and very high endemic areas such as France and Tunisia, respectively. Nevertheless, the prerequisite for these epidemiologic interpretations is the need for high genetic diversity in these areas, and a highly discriminating molecular tool.

Only a few studies using a sequencing approach have analyzed the genetic diversity of multiple CE cysts in a single host, and never in humans. Different haplotypes based on short *cox1* sequences of one cyst from the lungs and one from the liver in the same sheep and/or cattle were frequently identified in France and Moldova [29,30,31]. On this basis, Hidalgo et al. [32] recently identified from two to five different haplotypes of *E. granulosus* s.s. in a single bovine or sheep infected by two to twelve cysts from a highly endemic area in Chile, using sequencing of the full length of the *cox1* mitochondrial gene. Even though no associations between haplotype and cyst fertility, size, or adventitial layer characteristics were observed, the presence of more than two haplotypes in a single host suggested that animals might face successive infection events. Nevertheless, this type of analysis by classical sequencing of one gene has a low discriminatory power unless the number of targeted genes is increased considerably, which would be very fastidious, time-consuming, and expensive. The use of a fast and highly discriminatory molecular tool such as microsatellite testing appears to be relevant in this context. Microsatellites, also known as short tandem repeats (STRs) or simple sequence repeats (SSRs), are short sequences of non-coding DNA consisting of one to six nucleotide tandem repeats. They may be highly polymorphic and have specifically been used to explore the genetic diversity of *E. multilocularis* [33,34]. In a previous study, the two microsatellite sequences EgSca6 and EgSca11 identified from the *E. granulosus* s.s. genome revealed very high discriminatory power, similar to that obtained by sequencing a large part (8274 bp) of the mitochondrial genome, and confirmed the high genetic variability of *E. granulosus* s.s. observed in the larval stage [35]. Among the 75 *E. granulosus* s.s. cyst samples tested from France and Tunisia, 63 EgSca6-EgSca11 genotypes were observed, with 54 genotypes represented by only one sample and nine genotypes described in two to four samples. Given the high genetic variability of *E. granulosus* s.s. observed in this study, these microsatellites are expected to be discriminant enough to study the number of infection events through genetic diversity at the intra-individual host level, since an intermediate host would rarely be contaminated successively by the same microsatellite profile. However, the overall genetic diversity of *E. granulosus* s.s. in France is expected to be much lower than in Tunisia, due to the difference in the intensity of circulation of the parasite. Therefore, although this is probably a rare event, the frequency for an individual to be infected successively by the same microsatellite profile might be higher in France.

The aim of the present study was to describe the intra-individual genetic diversity of *E. granulosus* s.s. cysts using microsatellites in order to better understand CE infection patterns in humans and livestock, and in high and low endemic areas.

## 2. Results

### 2.1. Number of Cysts and Organ Localization per Host Species

**Figure 1:**
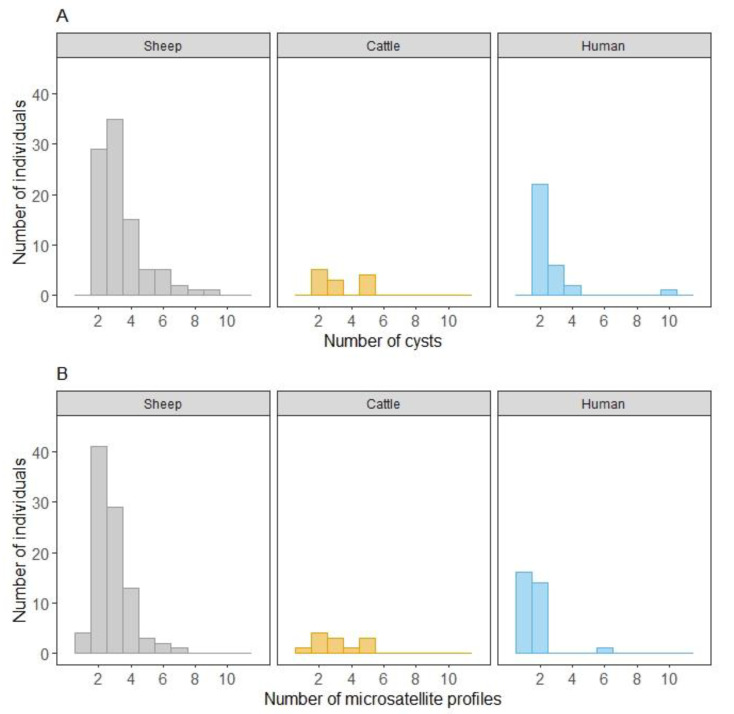
Distribution of the number of hosts according to the number of cysts (**A**) and to the number of microsatellite profiles (**B**) per host species (sheep, cattle and humans) for the panel of *E. granulosus* s.s. samples from France and Tunisia, screened by EgSca6 and EgSca11 microsatellites.

Among samples from sheep, cattle and humans, the number of cysts per host varied from two to ten (Supplementary Tables S1–S5), with a median of three (Table 1, Figure 1). The proportion of hosts carrying strictly more than three cysts was higher in cattle (33%, *n* = 12) and sheep (31%, *n* = 93) than in humans (10%, *n* = 31, Chi^2^ test, *p* < 0.001, Figure 1). In sheep, the median number of cysts sampled per animal was not different between the two countries of origin (Mann–Whitney U test, *p* = 0.39).

CE cysts were identified in two different organs for the same individual for 45 sheep (48%, *n* = 93), none for cattle (0%, *n* = 12), and 13 for patients (42%, *n* = 31). In humans, cysts were reported from the liver and lungs for nine patients, and from the spleen associated with the liver or the lungs for four children and one child, respectively. In sheep, the proportion of animals with cysts in both the liver and lungs was higher in the sample from France (71%, *n* = 42) than from Tunisia (*n* = 29%, *n* = 51, Chi^2^ test, *p* = 0.02).

### 2.2. Microsatellite Genetic Diversity in a Single Host According to Host Species and Country

Simpson Diversity Index values were 0.998 for Tunisia and 0.993 for France, indicating similar and very high genetic diversity in both countries. These index values show that the majority of EgSca6-EgSca11 profiles was observed in only one host (Supplementary Tables S1–S5).

In sheep, the number of different microsatellite profiles per host varied from one to four in France, while it reached seven profiles in Tunisia (Table 1, Figure 1). In all, 96% of sheep had at least two cysts with different microsatellite profiles, and 20% had from four to seven cysts with different microsatellite profiles. Only four sheep (4.3%, *n* = 93), one from France (#5239, 2.4%, *n* = 42) and three from Tunisia (#TunS6, #TunS25, #Tun36, 5.9%, *n* = 51), showed the same microsatellite profile for all their CE cysts (Table 1, Supplementary Tables S1 and S2). This proportion was not significantly different between the two countries (Fisher’s exact test, *p* = 0.6). The cysts of 33 sheep (35%, *n* = 93), 16 from France (38%, *n* = 42) and 17 from Tunisia (33%, *n* = 51), had at least two similar microsatellite profiles. According to generalized linear mixed model (GLMM) results (Table 2), the probability for a sheep to have at least two similar microsatellite profiles increased with the increased number of cysts per host, and was not influenced by the country of origin. Moreover, the Poisson GLM showed that the number of microsatellite profiles found per host increased linearly with the number of cysts, but did not depend on the country (R^2^ = 0.6). Two individuals were outliers: a sheep from France (#5239, Supplementary Table S1) with the same microsatellite profile for all of its seven cysts and a sheep from Tunisia (#TunS14, Supplementary Table S2) with nine lung cysts corresponding to four different microsatellite profiles.

Among the 12 infected cattle from Tunisia, one to five different microsatellite profiles were identified in a single host (Figure 1, Supplementary Table S3). Eleven (92%) harbored at least two different microsatellite profiles, and only two (17%) had at least two cysts with the same microsatellite profile. One with five liver cysts harbored four different profiles (#TunC7) and only one harbored the same profile for these two cysts (#TunC8).

In children, one to two different microsatellite profiles were found per patient, with the exception of one patient (#TunH30) harboring six different microsatellite profiles among the 10 cysts (Figure 1, Supplementary Tables S4 and S5). Overall, humans (*n* = 31) infected by multiple cysts with at least two similar or two different microsatellite profiles were observed for 20 (65%) and 15 (48%) individuals, respectively. One patient (#TunH23, Supplementary Table S4) with four lung cysts with the same microsatellite profile was operated on twice, with four years separating the first operation (three cysts) from the second (one cyst).

According to the GLMM results (Table 3), the odds for a human to have at least two cysts with the same microsatellite profile was 8.8 times higher [CI 95%: 3.2–26.1] than for a sheep, and increased with the number of cysts per host. No influence of the number of cysts (2 or ≥3) was found when including only human data in the logistic model. The probability for an individual to harbor at least two cysts with different microsatellite profiles was lower in humans than in sheep (OR of 0.1 [CI 95%: 0–0.3]) and was not influenced by the number of cysts.

The identification of the same profile in cysts that developed in different organs was observed only in five sheep (11% of sheep with cysts in two organs, *n* = 45) all originating from France and none in cattle (Table 1, Supplementary Tables S1–S3). In contrast, nine children (69% of the 13 patients with cysts in two different organs) harbored cysts with the same microsatellite profile (Table 1, Supplementary Table S4).

## 3. Discussion

The EgSca6 and EgSca11 microsatellites have proven to be useful when exploring the genetic diversity of *E. granulosus* s.s. at the intra-individual host level [35]. Due to the high mutation rate of microsatellites, they are potentially the most informative molecular markers, with much lower cost and easier data analysis than classical sequencing. They enable tracking of CE infections to estimate the single or multiple sources of infections of humans and intermediate hosts.

Multiple CE cysts are the result of the same or successive CE infection events (primary echinococcosis). In some cases, spontaneous or trauma-induced primary cyst rupture leading to spillage of protoscoleces and/or parasitic stem cells may lead to the development of new cysts in the same organ, or at another site in the body (secondary echinococcosis) [36]. Therefore, the presence of identical microsatellite profiles identified in a single host argues in favor of a single infection event (i.e., oral ingestion of one to several eggs at the same time) and/or secondary echinococcosis. However, two cysts with the same microsatellite profile may also result from two different infection events, and the probability of the occurrence of such events depends on the genetic diversity of *E. granulosus* s.s. in a given area. We initially hypothesized that this probability would differ between Tunisia and France. We assumed lower genetic diversity of *E. granulosus* s.s. in France than Tunisia, given that France has lower environmental contamination [29,37,38]. This hypothesis was assessed by comparing both the genetic diversity and the number of microsatellite profiles between sheep from France and Tunisia by controlling the number of cysts, expecting to find a difference if the frequency of animals infected by the same microsatellite profile differed between both countries. Unexpectedly, this number did not differ between the two countries, suggesting that the frequency of animals being infected successively by the same microsatellite profile was similar in both countries. Moreover, the genetic diversity of *E. granulosus* s.s. in our samples was very high and similar between the two countries, as suggested by the Simpson index values. This was even more unexpected given that the sheep sampled in France all originated from one endemic region in the south of the country, whereas those sampled in Tunisia were from different places and circulated widely in the country, increasing the probability of being infected with *E. granulosus* s.s. eggs of different microsatellite profiles. Therefore, our results indicate that the genetic diversity estimated by these microsatellites is high, even in a low endemic country such as France. Nevertheless, the genetic diversity in the south of France may be higher compared to other very low endemic areas, as the prevalence has drastically decreased in France only in recent decades. In conclusion, given the high genetic diversity in both countries, we considered that the cysts with the same microsatellite profile in a host resulted from the same infection event due to simultaneous oral ingestion of several eggs with the same microsatellite profile.

The presence of different microsatellite profiles in a single host can mainly be explained by successive infections of the host (i.e., successive oral ingestions of infective eggs with different microsatellite profiles). To a lesser extent, but with an unknown frequency, it could also be explained by a single infection with several *E. granulosus* s.s. eggs having different microsatellite profiles. For instance, this can occur when the eggs are in spatial proximity to each other in the environment (i.e., single oral ingestion of eggs), due to the simultaneous excretion of these eggs by a canid harboring several adult worms with different microsatellite profiles. However, there are no data available to evaluate the frequency of canids harboring worms with different microsatellite profiles and excreting eggs simultaneously. It is also unknown how many eggs develop into cysts when ingested together, but this number is thought to be low.

In our study, 35% of sheep had at least two cysts with the same microsatellite profiles. This suggests that a third of them ingested multiple eggs of the same microsatellite profiles simultaneously, which developed into two to seven cysts. Considering that the number of different microsatellite profiles is a proxy of the number of infection events, the proportion of infection events leading to the development of several simultaneous cysts among sheep with multiple CE cysts can be estimated as follows: the overall number of repeated microsatellite profiles within the same host (i.e., simultaneous developed cysts) for all sampled sheep divided by the overall number of microsatellite profiles present in all sampled sheep (Supplementary Table S1). This proportion was estimated to be 15% for the sheep in France. This estimate is, however, slightly overestimated since the overall number of infection events is probably lower than the overall number of different microsatellite profiles, given that in some cases, sheep can be simultaneously infested by eggs with different microsatellite profiles. A previous study conducted in the same area of the south of France highlighted that 45% of 93 sheep infected by *E. granulosus* s.s. were infected by at least two cysts (Umhang, unpublished data). This means that a maximum of 7% of infection events led to the development of several simultaneous cysts in sheep in France (100 × 45% of sheep with multiple cysts × 15% of events leading to the development of several simultaneous cysts among sheep with multiple CE cysts). Unfortunately, this estimate cannot be done for the Tunisian sheep due to incomplete slaughtering data. Additionally, 69% of the infection events by more than one egg (i.e., 11 infection events with two cysts with the same profile for a total of 16 infection events with two to seven cysts with the same profile) led to the development of only two cysts in sheep in France. A similar proportion in Tunisian sheep was found, which would indicate that the number of cysts developed after a single infection event might be similar between low and high endemic countries. However, these results need to be confirmed since the proportion in Tunisia might have been underestimated. In France, the cysts with the same microsatellite profile in a single host were mainly located in the same organ. This suggests that the development of cysts in two different organs after ingestion of several eggs from a single infection event is rare.

In the sheep samples, the number of microsatellite profiles at the intra-individual level varied from one to seven and increased linearly with the number of cysts sampled per animal. The great majority of sheep (96%) had at least two cysts with different microsatellite profiles, and 20% had from four to seven cysts with different microsatellite profiles. We cannot estimate from our results the average number of infection events that a sheep faced during its life, since the proportion of these cysts that are from the same infection event is unknown. However, because we previously estimated that only a low proportion of infection events (≤7%) leads to the development of several simultaneous cysts, our results suggest that most sheep with at least two cysts (unknown but estimated >80%) have faced multiple infections. As 45% of sheep in France are infected by at least two cysts and considering that 80–96% of sheep with at least two cysts in our study were infected at least twice, around 35–43% of sheep in southern France would face multiple infection events of *E. granulosus* s.s. (100 × 45% of sheep with multiple cysts × 80–96% of sheep with at least two different microsatellite profiles and infested at least twice). These results indicate that even in a very low endemic area (0.01% in sheep from this region [29]), multiple infections occur frequently. The number of cysts and microsatellite profiles per host was not significantly different between sheep from France and those from Tunisia. However, since not all the cysts were sampled in Tunisia, these two values are expected to be much higher in Tunisia than those measured in this study, with multiple infections occurring more frequently than in France.

In this study, only 12 cattle samples from Tunisia were analyzed. The number of CE cysts in single cattle was similar to that of in sheep. Like sheep, most cattle (11/12) had at least two cysts with a different microsatellite profiles, suggesting that they frequently faced successive infection events. However, unlike sheep, only two of them had at least two cysts with a similar microsatellite profile. Further investigations are needed to confirm whether such a difference exists, which would suggest that the development of multiple cysts after a single infection event is rarer in cattle than in sheep in Tunisia. Of note, cattle in North Africa are considered important intermediate hosts for *E. granulosus* s.s., with infections frequently leading to fertile cysts [14]. On the contrary, in Europe, for instance in France, cattle are considered unsuitable intermediate hosts due to the extremely low cyst fertility classically observed [13,29]. As a result, the data provided from cattle in this study need to be restricted to the North African epidemiologic context.

In the children in our study, 65% harbored multiple cysts with at least two similar microsatellite profiles. With a similar calculation as for sheep, we estimated that the maximum proportion of infection events leading to the development of several simultaneous cysts among children with multiple CE cysts was 42% (Supplementary Tables S4 and S5). As 20% of children in Tunisia are infected by two cysts or more, this means that the maximum proportion of infection events leading to the development of several simultaneous cysts was 8% in children (100 × 20% children with multiple cysts × 42% of events leading to the development of several simultaneous cysts among children with multiple CE cysts). This proportion is similar to that observed in French sheep. These events might have led to the development of two to four CE cysts per child, restricted to only two cysts in 70% of the cases. Interestingly, these results are again similar to those in sheep, which suggests that the number of cysts developed after a single infection event is not different between sheep and humans. Contrary to what was observed in sheep, 69% of patients with cysts with the same microsatellite profiles harbored them in two different organs. This suggests that development of cysts in two different organs after ingestion of several eggs from a single infection event is frequent in humans, but not in sheep. The reasons for these differences are currently unknown and all these results should be investigated further.

The infected children in our study harbored only two to four CE cysts with one to two different microsatellite profiles, except for one child with ten cysts and six different microsatellite profiles. The overall number of cysts and the number of microsatellite profiles in children was much lower than in livestock, as expected. Humans, as accidental hosts, are less exposed to CE than livestock due to the contamination pathway, ingestion of *E. granulosus* s.s. eggs via vegetables or water, and following direct contact with dogs, soil or fomites [39]. Previous studies have reported that in high endemic areas such as Tunisia, numerous pediatric CE cyst infections occur in about 20% of cases [27,28]. In this study, 48% of children harbored multiple cysts with at least two different microsatellite profiles. This indicates that most of these children have faced multiple infections, since only a few infection events (≤8%) lead to the development of several simultaneous cysts in children. Therefore, the real proportion of children with multiple cysts that have been infected several times is unknown, but is estimated at >30%. This means that successive CE infection events are not negligible in high endemic areas, occurring in around 5–10% of children (100 × 20% of children with multiple cysts × 30–48% of children harboring multiple cysts and infested at least twice). Moreover, considering that exposure to *E. granulosus* s.s. eggs continues throughout life, and that CE infection can remain latent over years or decades before triggering clinical symptoms, it is expected that adults might be more exposed to successive infections and harbor more microsatellite profiles than children. Unfortunately, no information about the frequency of multiple cysts in adults is currently available in Tunisia that could confirm this hypothesis.

In the management of CE disease, recurrence remains one of the major problems associated with the surgical removal of primary cysts [40,41]. Recurrence is defined as the appearance of a new growing cyst, undetected by imagery before the first surgery or by the surgeon during the first procedure [42]. In our series, the interval between two surgeries in the same patient is generally between 1 month and 1 year. For the vast majority of patients, the cysts of the second surgery were previously detected by imagery but not resected, and a maximum of two different microsatellite profiles were observed. These results suggest that these multiple cysts are due to one to two infection events before the first surgery. For one patient with four lung cysts with the same microsatellite profile (#TunH23), four years separate the first operation (three cysts) from the second (one cyst). The cyst of the second operation was detected neither by imagery (conventional radiography and computerized tomography) during the first diagnosis, nor during the surgical procedure. Considering the high genetic diversity observed using the EgSca6 and EgSca11 microsatellites, it also appears quite unlikely that this fourth cyst was the result of a second infection event with the same profile occurring during the four years separating the two surgical interventions. According to these data, this fourth cyst is considered to be a consequence of secondary CE due to unintentional protoscoleces spillage during the first operation or spontaneous/traumatic cyst rupture before the second interventional approach. Nevertheless, we cannot entirely rule out that this cyst could be the result of a very small cyst that was unnoticed during the first imagery. To our knowledge, this phenomenon of secondary CE previously described has never been assessed by a genetic approach.

## 4. Materials and Methods

### 4.1. Ethics Statements

Cysts isolated from animals were collected during postmortem examination following inspection by the veterinary officer at the slaughterhouses, with due consent and in the context of an official slaughterhouse survey in France. Human CE cyst sampling was approved by the ethics committee of the High Institute of Biotechnology of Monastir (CER-SVS-011/2020, ISBM, Tunisia).

### 4.2. Isolate Sampling

A total of 348 CE cyst samples from sheep and cattle from France and Tunisia and 80 from Tunisian children were studied (Table 1). Parasitic material from France consisted of a total of 133 cysts from the lungs and liver from 42 sheep (two to seven cysts per host) slaughtered in the south of France (Provence-Alpes-Côte d’Azur) during a CE survey in 2009–2010 [31] and 2012 [29]. Parasitic material from Tunisia consisted of 176 cysts from the lungs and liver from 51 sheep (two to nine cysts by host), and 39 from 12 cattle (two to five cysts by hosts) acquired from slaughterhouses of different localities throughout the country (Gafsa, Kairouan, Kasserine, and Sousse). In Tunisia, given the high number of cysts per organ (up to 50 cysts may develop in the same organ), not all the cysts per animal were collected [28]. Moreover, when the livestock organs (liver and lungs) were both infected, it was sometimes not mentioned whether they came from the same animal or not.

In addition, human parasitic material consisted of 80 cysts from the lungs, liver and spleen from 31 children aged 5–12 years (two to ten per individual) operated on at Monastir University Hospital. For each patient, the cysts were collected during one or two surgeries separated by one month to four years.

Protoscoleces or germinal layers were removed from CE cysts and kept frozen prior to molecular analysis. All samples used in this study were previously confirmed to be due to *E. granulosus* s.s. using PCR amplification of the mitochondrial 12S rRNA gene [43] and/or partial sequencing of the *cox1* gene [44].

### 4.3. PCR Amplification, Microsatellite Fragment Size Analyses and Profile Interpretation

Total DNA of isolates from Tunisia was extracted from the protoscoleces using the phenol/chloroform protocol proposed by Sambrook [45], subsequent to enzymatic digestion by proteinase K (Invitrogen, Carlsbad, CA, USA) for 1 h at 56 °C. DNA of isolates from France was extracted directly from the protoscoleces if present or from the germinal layer using an iPrep purification instrument (Invitrogen) and an extraction kit for tissue (Invitrogen, iPrep ChargeSwitch gDNA Tissue Kit, Carlsbad, CA, USA). Two highly polymorphic microsatellites EgSca6 and EgSca11 were used to analyze the genetic diversity of *E. granulosus* s.s. [35]. The microsatellites, located in non-coding regions of the *E. granulosus* s.s. genome, were composed of trinucleotide motif repetitions, (GAA) and (CCT) (CTT), respectively. The EgSca6 and EgSca11 microsatellites were amplified in a multiplex PCR and the products were submitted to capillary electrophoresis on a sequencer machine (GA3500, Life Technologies, Carlsbad, CA, USA) in order to determine the size and height of each peak of the microsatellite profiles.

The microsatellite profiles obtained for the CE cysts were compared for the same host (children and animals). Two cysts were considered as harboring two different microsatellite profiles (assumed to be due to allele values obtained for both targets) when at least one of the allele values was different. Genetic diversity was evaluated by calculating the Simpson Diversity Index [46], with the panel of 309 *E. granulosus* s.s. cysts in sheep from France and Tunisia. This index expresses the probability that two unrelated samples will be classified as genotypically different using the microsatellite analysis.

### 4.4. Statistical Analyses

In order to study the effect of host species (sheep, cattle, human) and country (France, Tunisia) on the intra-host diversity of microsatellite profiles, we analyzed the effect of these factors on (i) the probability for a host to have at least two cysts with different microsatellite profiles (which is equivalent to the inverse probability for a host to have all the cysts with the same microsatellites); (ii) the probability for a host to have at least two cysts with the same microsatellite profiles (which is equivalent to the inverse probability for a host to have all the cysts with different microsatellite profiles); and (iii) the probability for a host to have at least two cysts with the same microsatellite profile in two different organs. The corresponding proportions observed in our sample were calculated by species and by country and tested with the Chi^2^ test.

Then, the effect of the country on these probabilities was analyzed by modelling these probabilities using a binomial generalized linear model as a function of country and number of cysts per host. For this analysis, only the data collected on sheep were included, as this is the only species with data from France and Tunisia. In addition, the number of microsatellite profiles per individual was modelled using a Poisson GLM according to the country and the log-transformed number of cysts.

The effect of the host species (sheep, human) on these probabilities was analyzed by modelling these probabilities using the GLMM as a function of host species and number of cysts per host. Since the first analysis indicated that these probabilities were not influenced by the country (see Section 2.1), the data on sheep from both countries were included in the analysis. Cattle were removed from the analysis as the quantity of data was low.

Odds ratios and 95% confidence intervals were estimated. All statistical analyses were performed with R 3.5.0 software [47].

## 5. Conclusions

Studying the genetic diversity of *E. granulosus* s.s. at the intra-individual host level by using EgSca6 and EgSca11 microsatellites proved to be a useful approach to better understand infection events. Our results suggest that multiple infection events are frequent in livestock, even in a low endemic country such as France, and are less frequent but not negligible in children in a high endemic country such as Tunisia. They also indicate that in 7–8% of cases as a maximum, several infective eggs, from two to seven in our data, were ingested simultaneously and developed into cysts in ruminants and children. Moreover, this is the first time that genetic evidence of secondary CE was found. To better assess the number of infection events that livestock and humans faced, further studies appear necessary to confirm these results and to investigate the genetic diversity of adult worms in definitive hosts, which are responsible for environmental contamination.

## Figures and Tables

**Table 1:** *Echinococcus granulosus* sensu stricto cysts in livestock and humans from France and Tunisia.

Country of Origin	Host Species	Number of Cysts per Host	Number of Hosts	Number of Hosts Ranked by Number of Microsatellite Profiles Observed						
				1	2	3	4	5	6	7
France	sheep	2	15		15					
		3	15		7	8				
		4	7		1	4	2			
		5	1				1			
		6	3			1	2			
		7	1	1						
Tunisia	sheep	2	14	2	12					
		3	20	1	6	13				
		4	8			2	6			
		5	4			1	1	2		
		6	2					1	1	
		7	1						1	
		8	1							1
		9	1				1			
Tunisia	cattle	2	5	1	4					
		3	3			3				
		5	4				1	3		
Tunisia	humans	2	22	10	12					
		3	6	4	2					
		4	2	2						
		10	1						1	

**Table 2:** Results of the generalized linear mixed model (GLMM, logistic link function) of the probability for a sheep to have at least two cysts with a similar microsatellite profile. OR: odds ratio, 95% CI: 95% confidence intervals.

Variables	OR	95% CI	*p*
**Species (Ref = France)**			
Tunisia	0.5	0.2–1.4	0.2
**No. Cysts**	2.6	1.7–4.4	<0.001

**Table 3:** Results of the generalized linear mixed model (GLMM, logistic link function) of the probability for an infected host (sheep or human) to have all cysts with the same microsatellite profile. OR: odds ratio, 95% CI: 95% confidence intervals.

Variables	Probabilities for a Host to Have at Least Two Cysts with					
	Similar Microsatellite Profiles			Different Microsatellite Profiles		
	OR	95% CI	*p*	OR	95% CI	*p*
**Species (Ref = Sheep)**						
Human	8.8	3.2–26.1	<0.001	0	0–0.1	<0.001
**No. Cysts**	2.7	1.8–4.6	<0.001	1.1	0.7–1.7	0.74

## Supplementary Materials

The following are available online at https://www.mdpi.com/2076-0817/9/6/444/s1, Table S1: Values of the alleles from the EgSca6 and EgSca11 microsatellite profiles for each sheep cyst from France according to their organ of localization. The same microsatellite profile obtained for at least two cysts from the same host is indicated in bold. Table S2: Values of the alleles from the EgSca6 and EgSca11 microsatellite profiles for each sheep cyst from Tunisia according to their organ of localization. The same microsatellite profile obtained for at least two cysts from the same host is indicated in bold and in italics in the case of a second common profile. Table S3: Values of the alleles from the EgSca6 and EgSca11 microsatellite profiles for each cattle cyst from Tunisia according to their organ of localization. The same microsatellite profile obtained for at least two cysts from the same host is indicated in bold. Table S4: Values of the alleles from the EgSca6 and EgSca11 microsatellite profiles for each human cyst from Tunisia according to their organ of localization. The same microsatellite profile obtained for at least two cysts from the same host is indicated in bold. Table S5: Values of the alleles from the EgSca6 and EgSca11 microsatellite profiles for the human patient from Tunisia infected by ten cysts. The same microsatellite profile obtained for at least two cysts from the same host is indicated in bold, underlined or in italics.
